# MiR-26b/KPNA2 axis inhibits epithelial ovarian carcinoma proliferation and metastasis through downregulating OCT4

**DOI:** 10.18632/oncotarget.4363

**Published:** 2015-06-08

**Authors:** Jiaxin Lin, Lan Zhang, He Huang, Yongwen Huang, Long Huang, Jianhua Wang, Shuting Huang, Li He, Yun Zhou, Weihua Jia, Jingping Yun, Rongzhen Luo, Min Zheng

**Affiliations:** ^1^ State Key Laboratory of Oncology in South China, Guangzhou, P. R. China; ^2^ Collaborative Innovation Center for Cancer Medicine, Guangzhou, P. R. China; ^3^ Department of Gynecology, Guangzhou, P. R. China; ^4^ Department of Pathology, Sun Yat-Sen University Cancer Center, Guangzhou, P. R. China; ^5^ Department of Oncology, The Second Affiliated Hospital, Nanchang University, Nanchang, P. R. China; ^6^ Cardiovascular Department, Second People's Hospital of Guangdong Province, Guangzhou, P. R. China; ^7^ Department of Obstetrics and Gynecology, The First Affiliated Hospital, Sun Yat-Sen University, Guangzhou, P. R. China

**Keywords:** epithelial ovarian cancer, KPNA2, miR-26b, OCT4

## Abstract

Karyopherin alpha 2 (KPNA2) is a nuclear transport protein upregulated in many cancers. Our previous study has identified KPNA2 overexpression in epithelial ovarian carcinoma (EOC) tissues, which predicts poor prognosis. However, the mechanism of KPNA2 overexpression in EOC remains unclear. This study aimed to examine the role of miRNA in KPNA2 dysregulation. Our results showed that miR-26b was downregulated in EOC samples, and correlated inversely with KPNA2 expression. Low expression of miR-26b was associated with advanced FIGO stage, poor differentiation, higher risk of distant metastasis and recurrence. Downregulation of miR-26b predicted poor disease-free survival and overall survival in EOC patients. KPNA2 was validated as a direct target of miR-26b. Knockdown of KPNA2 or ectopic expression of miR-26b could downregulate OCT4, vimentin and upregulate E-cadherin. Reintroduction of KPNA2 partially abrogated the suppression effect induced by miR-26b. We further verified that miR-26b/KPNA2/OCT4 axis inhibited EOC cell viability, migratory ability and sphere-forming capacity *in vitro* and *in vivo*. In conclusion, our results reveal that miR-26b is downregulated in EOC, and directly targets KPNA2. miR-26b/KPNA2 axis suppresses tumor proliferation and metastasis through decreasing OCT4 expression, which is indicative of the important role of miR-26b/KPNA2/OCT4 axis in EOC carcinogenesis and progression.

## INTRODUCTION

Epithelial ovarian carcinoma (EOC) is the most common histological type of ovarian cancer, representing the most fatal gynecological malignancy among women worldwide. It was estimated that 21,980 new cases and 14,270 deaths occured in 2014 as a consequence of EOC [1]. The main reason for the high mortality is the lack of diagnostic methods for early stage detection and effective strategies for treatment. Therefore, understanding the molecular pathogenesis and uncovering molecular biomarkers of EOC would facilitate early detection and improve the survival of ovarian cancer patients.

By using cDNA microarrays, we previously found that karyopherin α2 (KPNA2) was overexpressed in EOC tissues compared to paired normal human ovarian surface epithelial (HOSE) tissues. Subsequent studies have shown that the overexpression of KPNA2 was correlated with a poor prognosis in EOC [2] and ovarian malignant germ cell tumors [3]. Additionally, we observed that KPNA2 could promote cell proliferation and tumorigenicity by enhancing c-Myc expression and reducing FOXO3a expression in EOC, suggesting that KPNA2 might be a potential therapeutic target in EOC [4].

Karyopherin α2 (KPNA2), a member of the importin α family, is thought to be an important part of nucleocytoplasmic transport [5-7]. Many recent clinical studies have demonstrated that KPNA2 is upregulated in multiple malignancies and associated with an adverse outcome for the affected patients [8-17]. The biological functions of KPNA2 have been examined in some cancer cell lines; for example, overexpression of KPNA2 in a benign breast cell line could increase cell colony formation ability and migration activity in a manner similar to that in malignant cells [18]. Cell migratory ability and viability can also be enhanced by KPNA2 in lung cancer cell lines [13]. Additionally, knockdown of KPNA2 inhibited the proliferation of cells derived from prostate [14], liver cancer [15] and ovarian cancer [4]. KPNA2 could promote carcinogenesis mainly through the translocation of cancer-associated cargo proteins, including proteins with tumor-suppressive or oncogenic properties. Previous evidence suggested that the nuclear localization of OCT4 is mediated by KPNA2 in mouse ES cells during differentiation into neurons [19]. It's also confirmed that OCT4 and KPNA2 have a strong interaction in ES cells [20]. OCT4 and KPNA2 play an important role in non-small-cell lung cancer progression: reduction of KPNA2 expression significantly reduces mRNA and nucleoprotein levels of OCT4. Moreover, knockdown of OCT4 or KPNA2 expression inhibits lung cancer cell proliferation [21].

OCT4 (also known as OCT3/4), encoded by the *Pou5f1* gene, belongs to the POU transcription factor family [22]. OCT4 is pivotal in maintaining self-renewal and pluripotency of embryonic stem (ES) cells [23]. It is well documented that OCT4 is also highly expressed in many cancer cell types [24], and leads to tumorigenicity, tumor metastasis and recurrence after chemoradiotherapy as a cancer stem cells marker [25]. The first report regarding OCT4 in EOC stem cells demonstrated that the tumorigenic clones were isolated from the ascites of a patient with advanced EOC, which displayed heterogeneity in their clonogenic, tumorigenic, and invasive properties and also exhibited stem and progenitor-like characteristics [26]. A recent study has demonstrated that a sub-population of human EOC cells expresses both Lin28 and OCT4, and this co-expression pattern is associated with increased tumor grade. Additionally, their combined repression results in synergistic inhibition of EOC cell growth and survival [27]. These studies suggest that OCT4 may be one of the defining features of ovarian cancer stem cells regulating cancer initiation and progression.

Many recent findings have indicated that microRNAs (miRNAs) serve significant functions in almost every biological pathway [28-32], and that abnormal miRNAs expression is associated with human cancers [30, 33-35]. miRNAs are a class of endogenous small (18–24 nt) noncoding single-stranded RNAs that regulate gene expression on post-transcriptional level by binding to the 3′-untranslated region (3′-UTR) of messenger RNA (mRNA), leading to mRNA degradation or the suppression of protein translation [36]. Some studies have demonstrated the dysregulation and function of miR-26b in diverse types of cancers [37-42]. Additionally, existing researches showed that miR-26b accelerates neuronal cell differentiation [43] and osteogenic differentiation of unrestricted somatic stem cells [44]. Human embryonic stem cells and metastatic colorectal cancer cells share the common endogenous human miR-26b. Overexpression of miR-26b leads to the inhibition of cell growth *in vitro* and *in vivo* [45]. However, the functional role of miR-26b in EOC has never been reported, and expression data for miR-26b in EOC from microarray profiling has yielded contradictory and controversial findings [46-48]. What's more, the role of KPNA2 together with miR-26b in relation to OCT4 remains obscure.

Although KPNA2 plays an important role in cancers, limited information is available regarding factors that control its expression. Therefore, the molecular mechanisms underlying KPNA2 overexpression remain to be further elucidated. In order to explore the mechanism of KPNA2 dysregulation in EOC, in this study we explored whether miRNAs could suppress KPNA2 expression, and sought to identify the biological behaviors that they affect in EOC cells. Here, we reported that miR-26b expression was repressed in EOC samples, and correlated inversely with KPNA2 expression. Downregulation of miR-26b was associated with aggressive and poor prognostic phenotype in EOC patients. KPNA2 was identified as a direct target of miR-26b. Knockdown of KPNA2 or ectopic expression of miR-26b could markedly enhance endogenous E-cadherin levels, while reduce expression of OCT4 and vimentin. Reintroduction of KPNA2 partially abrogated the suppression effect induced by miR-26b. We further verified that miR-26b/KPNA2/OCT4 axis inhibited EOC cell growth, metastasis and sphere formation *in vitro* and *in vivo*.

## RESULTS

### An inverse correlation between KPNA2 and miR-26b expression in EOC

Our previous studies have shown that KPNA2 is overexpressed in epithelial ovarian cancer and correlates with a poor prognosis [2]. In this study, we aimed to explore whether miRNAs are associated with KPNA2 expression.

**Figure 1:**
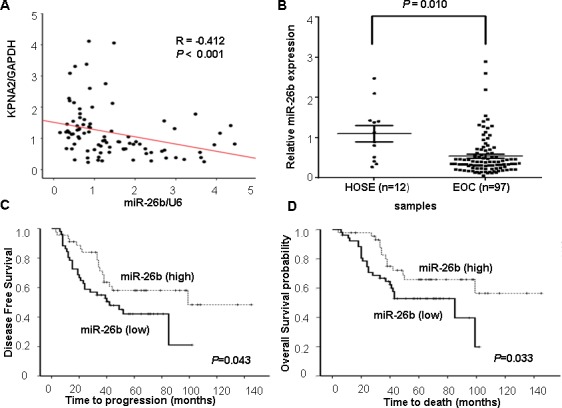
miR-26b expression and its inverse correlation with KPNA2 expression in EOC **A.** The correlation between miR-26b and KPNA2 expression in EOC tissue samples (*n* = 93; four samples with outlying values were excluded). y, the relative expression of *KPNA2* mRNA; *GAPDH* mRNA was used as an internal control. x, the relative levels of miR-26b; U6 was used as an internal control. Correlations were performed using Pearson's correlation analysis. **B.** Relative miR-26b expression in 12 HOSE (normal human ovarian surface epithelial tissues) and 97 EOC (epithelial ovarian carcinoma) was determined by qRT-PCR. (**C**, **D**) Correlations between miR-26b levels and both disease-free survival and overall survival were tested based on Kaplan-Meier analysis in patients with high (*n* = 48) or low miR-26b expression (*n* = 49); *P* < 0.05.

We initially integrated three computational algorithms, including miRanda, PicTar and TargetScan, to search for miRNAs that target the 3′-UTR of *KPNA2* mRNA. According to these prediction analyses, the miR-26b-binding site mapped correspondingly to the *KPNA2* 3′-UTR. Thus, miR-26b was of particular interest to us. To determine whether expression of KPNA2 was associated with miR-26b, we examined the levels of KPNA2 and miR-26b in 93 EOC specimens and observed a moderate inverse correlation (R = −0.412; *P* < 0.05; Figure 1A).

Next, miR-26b expression was measured in 97 human EOCs and 12 HOSE tissues by qRT-PCR. We found that miR-26b was significantly underexpressed in EOC compared with HOSE (*P* < 0.05; Figure 1B). To assess the clinical significance of miR-26b levels, patients were separated into the miR-26b ‘high-level’ (*n* = 48) and ‘low-level’ (*n* = 49) groups according to the median value of miR-26b expression. As shown in Table 1, low miR-26b levels was correlated with advanced FIGO (the International Federation of Gynecology and Obstetrics) stage (*P* = 0.003), poor differentiation (*P* = 0.021), higher risk of distant metastasis and recurrence (*P* = 0.032). Kaplan-Meier survival analysis showed that ‘low-level’ miR-26b group was associated with poor disease-free survival and overall survival in EOC patients (*P* < 0.05; Figure 1C, 1D). Moreover, differentiation, the FIGO stage and miR-26b expression were associated with overall survival, as indicated by univariate analysis. However, multivariate analysis indicated that differentiation and the FIGO stage were independent prognostic factors for EOC patients, independent of miR-26b expression (Table 2).

**Table 1:** Correlations between miR-26b expression and clinicopathological features of patients with epithelial ovarian cancer

Characteristics	miR-26b expression		
	High (%)	Low (%)	*P*-value
*Age, years*			
< 45	12	16	0.406
≤ 45	36	33	
*FIGO stage*			
I/II	25	11	**0.003**
III/IV	23	38	
*Differentiation*			
Well	10	5	**0.021**
Moderate	17	12	
Poor	13	28	
Missing data	8	4	
*Distant metastasis and recurrence*			
Yes	31	21	**0.032**
No	17	28	

Bold *P*-values indicate significance (*P* < 0.05)

**Table 2:** Univariate and multivariate analysis of factors associated with overall survival

Clinical variable	Subset	Hazard ratio (95% CI)	*P*-value
***Univariate analysis***			
FIGO stage	III/IV versus I/II	1.730 (1.249–2.397)	0.001
Differentiation	Poor versus moderate versus well	5.095 (1.767–14.695)	0.003
Expression of miR-26b	High versus low	0.479 (0.247–0.929)	0.03
***Multivariate analysis***			
FIGO stage	III/IV versus I/II	1.980 (1.266–3.097)	0.003
Differentiation	Poor versus moderate versus well	3.419 (1.312–8.905)	0.012

**Abbreviations: CI,** confidence interval; **FIGO**, International Federation of Gynecology and Obstetrics.

### KPNA2 is a direct target of miR-26b

**Figure 2:**
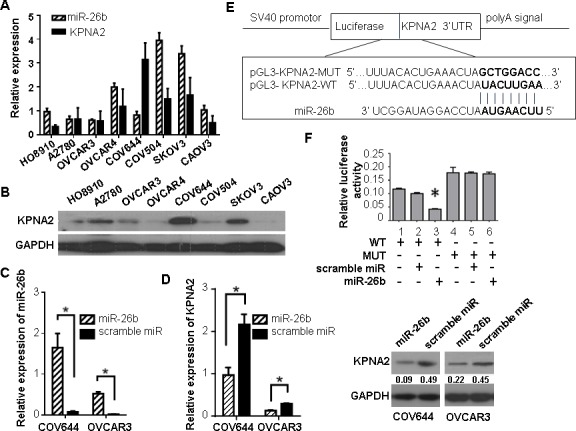
KPNA2 is a direct target of miR-26b **A.** The relative *KPNA2* mRNA (normalized to *GAPDH* mRNA), and miR-26b (normalized to U6) expression were detected by qRT-PCR in eight EOC cell lines. **B.** Relative KPNA2 protein (normalized to GAPDH) levels were measured by western blotting in eight EOC cell lines. **C.** The levels of miR-26b in COV644 and OVCAR-3 cells significantly increased after transfection with miR-26b mimics, as compared to cells transfected with a scramble miRNA. **D.** Repression of KPNA2 expression at the post-transcriptional (top) and translational (bottom) levels in COV644 and OVCAR-3 cell lines treated with miR-26b mimics. **E.** The binding sites of miR-26b in the KPNA2 3′-UTR are shown. The 3′-UTR of *KPNA2* mRNA (pGL3-KPNA2-WT) and a mutant variant (pGL3-KPNA2-MUT) were constructed and cloned into a pGL3-control luciferase reporter vector. **F.** Relative luciferase activity in 293T cells was determined after the WT or MUT 3′-UTR of KPNA2 plasmids were co-transfected with miR-26b mimics or scrambled miRNA. All data are presented as the means ± SD obtained from three independent experiments; * *P* < 0.05.

We further explored the inverse correlation between KPNA2 and miR-26b expression in eight EOC cell lines (A2780, HO8910, COV504, COV644, OVCAR-3, OVCAR-4, CAOV-3 and SKOV3). In most of the EOC cell lines, both the mRNA and protein levels of KPNA2 were negatively correlated with miR-26b expression (Figure 2A, 2B). COV644 and OVCAR-3, which showed high levels of KPNA2 expression and low levels of miR-26b expression, were transfected with miR-26b mimics. As measured by qRT-PCR and western blotting, ectopic overexpression of miR-26b was observed to suppress endogenous KPNA2 expression at both the mRNA and protein levels when compared to cells transfected with scramble miRNA (*P* < 0.05; Figure 2C, 2D).

To characterize whether miR-26b suppresses KPNA2 expression by directly targeting the 3′-UTR, a luciferase assay on 293T and OVCAR-3 cell lines was performed. The 3′-UTR of KPNA2 mRNA (pGL3-KPNA2-WT) and a mutant variant (pGL3-KPNA2-MUT) were constructed and cloned into a luciferase reporter vector (pGL3-control; Figure 2E). When pGL3-KPNA2-WT was co-transfected with miR-26b, we found a significant reduction in luciferase activity compared to cells transfected with the scramble miRNA (*P* < 0.05, Figure 2F). However, no difference in luciferase activity was observed in cells co-transfected with pGL3-KPNA2-MUT and miR-26b. Our findings strongly suggest that KPNA2 expression is suppressed by miR-26b through directly binding.

### Repression of KPNA2 expression by miR-26b inhibits EOC cell growth, migration and sphere-forming capacity *in vitro*

**Figure 3:**
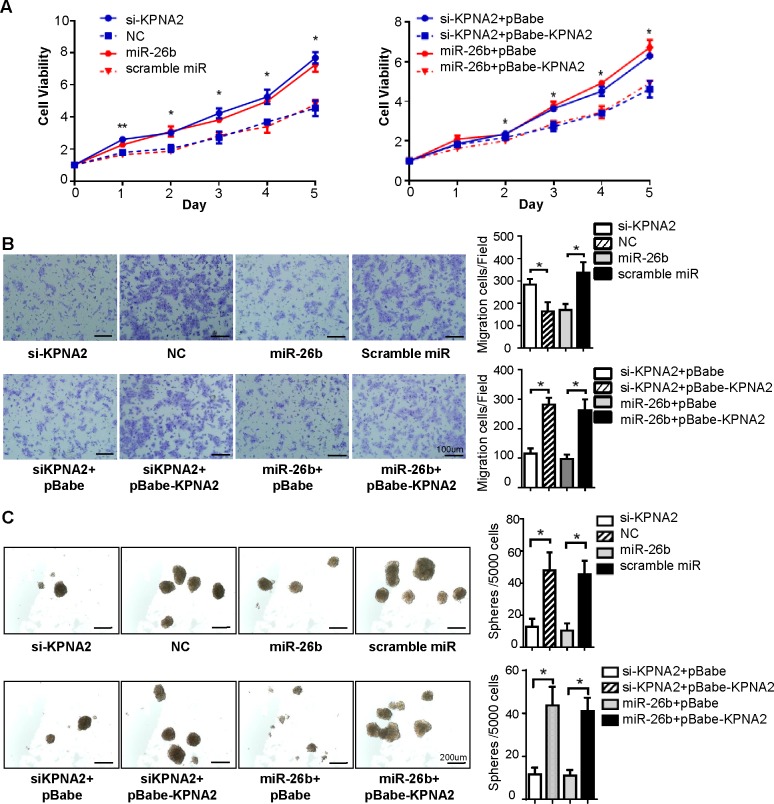
Upregulation of KPNA2 induced by miR-26b reduction promotes EOC cell growth, migration and sphere-forming capacity *in vitro* A representative MTT assay **A.**, transwell migration assay **B.** and sphere formation assay **C.** were performed using the OVCAR-3 cell line. Knockdown of *KPNA2* or ectopic expression of miR-26b could significantly inhibit cell viability (*P < 0.05), migratory ability (*P < 0.05) and sphere-forming capacity (*P < 0.05) compared to that of cells transfected with NC/scramble miRNA. Reintroduction of KPNA2 partially abrogated the suppression effect induced by miR-26b (*P < 0.05). The statistical results of the tumor spheres ( >50 μm) were calculated. The scale Bars in figure B and C respectively represent 100 μm and 200 μm. All data are representative of three independent experiments.

**Figure 4:**
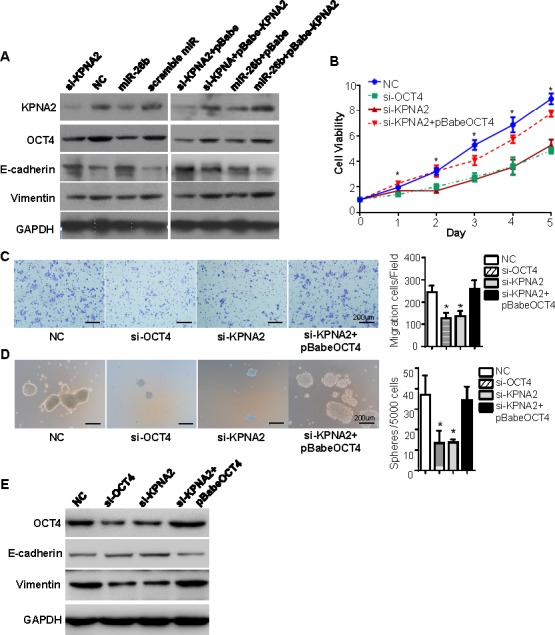
miR-26b/KPNA2 inhibits EOC cell growth, migration and sphere-forming capacity through decreasing OCT4 expression *in vitro* **A.** Western blot analysis revealed that miR-26b mimics could decrease the expression of KPNA2, OCT4, vimentin, and increase E-cadherin levels, similar to the effect of KPNA2 siRNA. The changes of KPNA2, OCT4, vimentin, E-cadherin expression were rescued in OVCAR-3 cells that were transfected with KPNA2 siRNA or miR-26b mimics, followed by pBabe-KPNA2. Knockdown of KPNA2 or OCT4 expression remarkably reduced the viable cells **B.** as well as migratory cells **C.**, and formed fewer, smaller spheres **D.**. The suppression effects by si-OCT4 and si-KPNA2 were abrogated followed by OCT4 reintroduction. **E.** Western blotting was used to detect the protein levels of OCT4, vimentin and E-cadherin in OVCAR-3 cells treated with knockdown of KPNA2 or OCT4 expression, or followed by OCT4 reintroduction. The scale Bars in figure C and D both represent 200 μm. All experiments were performed in triplicate, and some results are shown as the mean ± standard deviation. *P < 0.05.

To investigate how miR-26b/KPNA2 axis alters biological behaviors in EOC cells, cell proliferation assay, migration assay and sphere formation assay for the OVCAR-3 cell line were designed. Firstly, overexpression of miR-26b was used to verify that the suppressive effect of miR-26b could mimic that of the reduced expression of KPNA2 (Figure 4A). We found that knockdown of *KPNA2* or ectopic expression of miR-26b could significantly inhibit cell viability (*P* < 0.05; Figure 3A), migratory ability (*P* < 0.05; Figure 3B) and sphere-forming capacity (*P* < 0.05; Figure 3C) when compared to cells transfected with NC/scramble miRNA.

Subsequently, we tested whether ectopic expression of KPNA2 could rescue the effect of miR-26b. Cells were transfected with *KPNA2* siRNA or miR-26b mimics, and followed by pBabe-KPNA2 (encoding the full-length coding sequence of *KPNA2* without the 3′-UTR region). KPNA2 levels were again elevated after overexpression of KPNA2 in cells transfected with *KPNA2* siRNA or miR-26b mimics (Figure 4A). Reintroduction of KPNA2 partially abrogated the growth arrest, migratory suppression and sphere-forming inhibition induced by miR-26b (*P* < 0.05; Figure 3A, 3B, 3C). Western blot analysis showed that si-KPNA2 or miR-26b mimics reduced E-cadherin expression and increased vimentin levels compared with the negative control counterpart (Figure 4A), and the above changes in EMT markers (E-cadherin, vimentin) expression were revoked after KPNA2 reintroduction. This data further proved that miR-26b inhibited EOC cell migration through decreasing KPNA2 expression. Based on our findings therewith, we concluded that the overexpression of KPNA2 caused by the suppression of miR-26b promotes cell proliferation, migration and sphere formation in EOC.

### miR-26b/KPNA2 inhibits EOC cell growth, migration and sphere-forming capacity through decreasing OCT4 expression *in vitro*

Given OCT4 is translocated by KPNA2 [19-20], we explored whether miR-26b/KPNA2 axis decreases OCT4 expression to inhibit EOC cell growth, migration and sphere-forming capacity. As shown in Figure 4A, OCT4 was downregulated by *KPNA2* siRNA or miR-26b mimics. We then tested cell viability, migratory ability and sphere-forming capacity in the cells treated with si-OCT4 or si-KPNA2. Compared to the negative control, knockdown of KPNA2 or OCT4 expression remarkably reduced the number of viable cells (*P* < 0.05; Figure 4B) as well as migratory cells (*P* < 0.05; Figure 4C), and formed fewer, smaller spheres (*P* < 0.05; Figure 4D). However, the suppression effects by si-OCT4 and si-KPNA2 were abrogated followed by OCT4 reintroduction (*P* < 0.05; Figure 4B, 4C, 4D).

Consistently, both si-OCT4 and si-KPNA2 effectively attenuated the protein levels of OCT4 and vimentin, while enhanced E-cadherin expression (Figure 4E). In the cells transfected with si-KPNA2 plus pBabe-OCT4, the levels of OCT4 was rescued, with increased vimentin and decreased E-cadherin (Figure 4E).

These data supported that miR-26b/KPNA2/OCT4 axis could inhibit EOC cell proliferation, migration and sphere-forming ability *in vitro*.

### miR-26b/KPNA2/OCT4 axis inhibits EOC growth and metastasis *in vivo*

**Figure 5:**
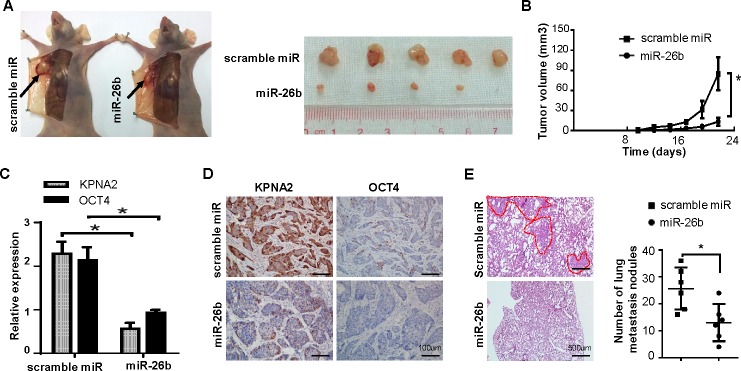
miR-26b/KPNA2/OCT4 axis inhibits EOC growth and metastasis *in vivo* **A.** Representative pictures of xenografts tumor model constructed by subcutaneously injecting OVCAR-3 cells stably overexpressing miR-26b or scrambled miRNA in the dorsal flank of nude mice, and illustration of subcutaneous tumors excised from the mice mentioned above (*n* = 5). **B.** The tumor growth rate of the experimental mice measured by tumor volumes (*P < 0.05). All data are shown as mean ± S.D. qRT-PCR and IHC were respectively used to detect the mRNA **C.** and protein **D.** level of KPNA2 and OCT4 in the mice tumors from miR-26b overexpressing group and control group. The scale bars represent 100 μm. **E.** Representative results for H&E staining of the metastatic nodules in the lung are shown. The metastatic nodules are indicated with dotted line. Bars represent 500 μm. The statistical result is on the right. *n* = 6; *P < 0.05.

To determine whether miR-26b was responsible for EOC tumorigenicity, we subcutaneously injected OVCAR-3 cells stably overexpressing miR-26b or scrambled miRNA into the dorsal flank of nude mice. Compared with control, the tumor volume and weight of the LV-miR26b group were markedly reduced (Figure. 5A, 5B; *P* < 0.05). As the results of qRT-PCR and IHC experiments respectively shown in Figure 5C, 5D, the mRNA and protein levels of KPNA2 and OCT4 were significantly lowered in tumors from the miR-26b overexpressing group (*P* < 0.05).

Furthermore, the cells were respectively injected into the tail vein of nude mouse to investigate the effect of miR-26b on metastasis. As shown in Figure 5E, the micrometastases of lung in LV-miR26b group were much fewer and smaller than that of the control group (*P* < 0.05).

In conclusion, miR-26b inhibited EOC growth and metastasis through decreasing the expression of KPNA2 and OCT4 *in vivo*.

## DISCUSSION

Published reports and our previous studies have established that KPNA2 is highly expressed in multiple malignancies [2, 3, 8-17], and its alterations are often associated with an adverse outcome for breast carcinomas [9-11], esophageal cancer [12], lung cancer [13], prostate cancer [14], hepatocellular carcinoma [15], bladder cancer [16], brain cancer [17] and ovarian cancer [2, 3]. However, the mechanism on how KPNA2 contribute to tumorigenesis and the development of carcinomas remains poorly understood. The biological functions of KPNA2 have been examined in some cancer cell lines; for example, overexpression of KPNA2 in a benign breast cell line could increase cell colony formation ability and migration activity in a manner similar to that in malignant cells [18]. Cell migratory ability and viability can also be enhanced by KPNA2 in lung cancer cell lines [13]. Additionally, knockdown of KPNA2 inhibited the proliferation of cells derived from lung [13], prostate [14], liver cancer [15] and ovarian cancer [4]. Limited data exists on the mechanism of dysregulation of KPNA2. Recently, Klf2 and Klf4 were shown to function redundantly to drive high levels of KPNA2 in mouse embryonic stem cells, which suggested that the regulation of *KPNA2* promoter activity is somewhat cell line-specific [49]. Some reports have shown that elevated Kpnβ1 and Kpnα2 expression in cancer cells correlate with dysregulated E2F/Rb activities [50]. Additionally, E2F1 is translocated in response to KPNA2, raising the possibility of a positive feedback loop. The elevated activity of E2Fs may lead to increased expression of KPNA2, which in turn increases the nuclear abundance of E2Fs [51]. Previously, we found that KPNA2 promotes cell proliferation and tumorigenicity through the upregulation of c-Myc and downregulation of FOXO3a in EOC, suggesting that KPNA2 may be a potential therapeutic target [4].

To explore whether KPNA2 expression in EOC is suppressed by miRNAs, we initially used three computational algorithms to search for miRNAs that target the 3′-UTR of *KPNA2* mRNA. We found miR-26b level is significantly lower in EOC, and correlated with FIGO stage, differentiation, distant metastasis, and prognosis of EOC patients. We then demonstrated that there is a moderate inverse correlation between the levels of KPNA2 and miR-26b, which was also confirmed both at the transcriptional and translational levels in the EOC cell lines. When EOC cells were transfected with miR-26b mimics, ectopic overexpression of miR-26b markedly suppressed KPNA2 expression at both the mRNA and protein levels when compared to cells transfected with scramble miRNA. We confirmed that KPNA2 is a direct target of miR-26b. Overexpression of KPNA2 caused by the suppression of miR-26b could significantly inhibit cell viability, migratory ability and sphere-forming capacity *in vitro.* Knockdown of KPNA2 or ectopic expression of miR-26b could downregulate OCT4, vimentin and upregulate E-cadherin. Reintroduction of KPNA2 partially abrogated the suppression effect induced by miR-26b. Additionally, si-OCT4 or si-KPNA2 remarkably reduced the viable cells as well as migratory cells, and formed fewer, smaller spheres. However, the suppression effects were revoked followed by OCT4 reintroduction. This findings support that miR-26b/KPNA2 suppresses EOC cell growth, migration and sphere formation through decreasing OCT4 expression. Furthermore, miR-26b/KPNA2/OCT4 was confirmed to inhibit EOC proliferation and metastasis *in vivo*.

Several lines of evidence from literature have provided relevant insight into the dysregulation and function of miR-26b in diverse cancers. In glioma, miR-26b directly reduces EphA2 expression and may act as a tumor suppressor [37]. In human breast cancer, downregulation of miR-26b can suppress cell apoptosis by targeting SLC7A11 [38] and promote cellular growth by binding to PTGS2 [39]. Additionally, miR-26b has also been shown to inhibit colorectal cancer cell growth and invasion, and this effect was partially abrogated by the addition of nicotinamide adenine dinucleotide [40]. Some reports have suggested that miR-26b can suppress NF-κB signaling and thereby sensitize hepatocellular carcinoma cells to the doxorubicin-induced apoptosis by inhibiting the expression of TAK1 and TAB3 [41]. Another study reported that miR-26 family members are concomitantly expressed with their host genes (CTDSP1/2/L), and cooperate to block the G1/S-phase transition by synergistically activating the pRb protein [42]. Contradictory expression data for miR-26b in EOC was reported in some microarray gene expression profiles [46-48]. Our study shows for the first time that miR-26b is significantly underexpressed and exerts its function by directly targeting KPNA2 in EOC.

Some limitations remain within the presented study. First, we were unable to detect obvious reduction in the levels of miR-26b when EOC cell lines were transfected with a miR-26b inhibitor (data not shown). It is possible that the background expression of miR-26b in EOC cells might be too low for such an effect to be observable. Second, the reintroduction of KPNA2 partially abrogated the growth arrest and metastasis suppression induced by miR-26b. As we known, miRNAs can have dynamic interactions with multiple target genes. Hence, other genes might be decreased by miR-26b, as KPNA2 overexpression could not completely counteract the effect of miR-26b. Furthermore, KPNA2 might also be modulated by other miRNAs or regulatory molecules; for instance, long non-coding RNA (lncRNA), a newly identified class of regulatory molecules, is involved in a variety of biological functions. Due to the complexity of control factors in gene expression networks, further investigations are needed to address these issues.

In summary, our studies were the first to identify miR-26b as downregulated and associated with aggressive and poor prognostic phenotype in EOC. KPNA2 is a target gene of miR-26b, and its overexpression in response to miR-26b reduction might promote tumor proliferation and metastasis through increasing OCT4 expression. This link between miR-26b, KPNA2 and OCT4 identifies a novel potential therapeutic target for the treatment of EOC.

## MATERIALS AND METHODS

### Human tissue samples and cell lines

For qRT-PCR experiments, snap-frozen tissues were obtained from 97 EOC patients who underwent surgery at Sun Yat-sen University Cancer Center (Guangzhou, China) between 2002 and 2010. All patients were histologically confirmed and did not receive chemotherapy. All specimens were obtained with the informed consent of patients, and this study was approved by the ethics committee of Sun Yat-sen University Cancer Center.

We obtained eight human EOC cell lines (A2780, HO8910, COV504, COV644, OVCAR-3, OVCAR-4, CAOV-3 and SKOV3) and the human embryonic kidney cell line 293T from the American Type Culture Collection. We cultured the cell lines in Dulbecco's Modified Eagle's medium (DMEM) with 10% fetal bovine serum (FBS; Invitrogen, Carlsbad, CA, USA).

### RNA isolation and quantitative real-time PCR analysis

Total RNA from tissues and cultured cells was extracted using Trizol (Invitrogen). Reverse transcriptase reactions and qRT-PCR were performed using the M-MLV reverse transcriptase (Promega, Madison, WI, USA) and Platinum SYBR Green qPCR SuperMix-UDG reagents (Invitrogen), respectively. The expression of miR-26b was detected using a Bulge-Loop™ miRNA qRT-PCR Primer Set (Ribobio, Guangzhou, China) according to the manufacturer's instructions. U6 was used as a reference. Primers were designed for *KPNA2* (forward, 5′-CTCATAACCATGTCCAC

CAACG-3′; reverse, 5′-CTCTATTCTGCGACGCCTCAT-3′) *OCT4* (forward, 5′- AGCACGAGTGGAAAGCAACT-3′, reverse, 5′-TTCTAGCTCCTTCTGCAGGG-3′), and *GAPDH* (forward, 5′-CTCCTCCTGTTCGACAGTCAGC-3′; reverse, 5′-CCCA ATACGACCAAATCCGTT-3′). *GAPDH* was used as an internal control. The relative fold changes in *KPNA2* mRNA and miR-26b expression were calculated using the 2−ΔΔ^Ct^ method.

### Plasmids

KPNA2 expression vector (pBabe-KPNA2) and OCT4 expression vector (pBabe-OCT4) were generated by respectively subcloning the full-length human KPNA2 and OCT4 cDNA into the pBABE-puro plasmid (Langri, GangZhou, China). pCDH-miR-26b were provided by Professor SM Zuang (Sun Yat-Sen University).

### Cell transfection

The hsa-miR-26b mimics and scramble oligonucleotides were obtained from Ribobio (Ribobio, Guangzhou, China). Human *KPNA2* siRNA (sc-35741) and *OCT4* siRNA (sc-36123) was purchased from Santa Cruz Biotechnology (Santa Cruz, CA, USA). COV644 and OVCAR-3 cell lines were seeded on 6-well plates and transfected the next day using Lipofectamine 2000 (Invitrogen), according to the manufacturer's instructions. Cells were harvested 48h after transfection for qRT-PCR analysis and western blotting.

### Western blot analysis

Total cellular proteins were extracted and separated on SDS-PAGE gels, and western blotting was performed in accordance with standard procedures (for details, please see reference 4). GAPDH was used as a loading control on the same membrane. The primary antibodies used include anti-KPNA2 (ab82313, Abcam) (sc-6917, Santa Cruz), anti-OCT4 (ab19857, Abcam), anti-E-cadherin (610181, BD), anti-Vimentin (5741, Cell Signaling Technology) and anti-GAPDH (sc-32233, Santa Cruz).

### Luciferase assay

A fragment of the 3′-UTR of *KPNA2* mRNA (region 1723-2011, from NM_002266) and a mutant variant were cloned into the *Xba*I site of a pGL3-control vector (Promega); the new vectors were named pGL3-KPNA2-WT and pGL3-KPNA2-MUT, respectively. 293T and OVCAR-3 cells were seeded at 5×10^4^ cells per well in a 24-well plate and co-transfected 24h later with pGL3-control (200 ng), pGL3-KPNA2-WT (200 ng), pGL3-KPNA2-MUT (200 ng), pGL4.73 vector (10 ng; Promega), miR-26b mimics (50 pmol) or scramble miRNA (50 pmol) using Lipofectamine 2000 (Invitrogen). Cells were harvested 48h after transfection and luciferase activities were analyzed using the Dual-Luciferase Reporter Assay System in accordance with the manufacturer's instructions (Promega). Firefly luciferase activity was then normalized to that of Renilla luciferase. All experiments were performed in triplicate.

### *In vitro* proliferation assay

An 3-(4,5-dimethylthiazole-2-yl)-2,5-biphenyl tetrazolium bromide (MTT) assay was used to detect cell proliferation. Briefly, cells (1500 cells/well) were seeded in 96-well plates. After transfection, the cells were cultured for the indicated time intervals and stained with sterile MTT dye (0.5 mg/ml, Sigma). After 4 h incubation, the supernatant was aspirated, and dimethyl sulfoxide (Sigma) was added. The absorbance was measured at 490 nm. All experiments were performed in triplicate.

### *In vitro* migration assay

Cell migratory potential was evaluated using transwell chambers (8 μm pore; BD Biosciences). A cell suspension (5×10^4^) in DMEM medium without serum was pipetted into the upper insert of a 24-well chamber 24 h after transfection; the bottom chamber was filled with DMEM containing 10% FBS. After 24h, cells remaining on the upper surface of the membrane were removed. Cells that had migrated to the lower surface of the membrane were stained with crystal violet and five independent fields were counted under a microscope. All experiments were performed in triplicate.

### Sphere formation assay

Per the method described by Dontu *et al*. [52] with minor modifiations, Single-cell suspensions were seeded at 5 × 10^3^ cells per well in ultra-low attachment 6-well plates (Corning, Lowell, MA, USA), and cultured with DMEM/F12 supplemented with 20 ng/mL epidermal growth factor (R&D Systems), 20 ng/mL basic ﬁbroblast growth factor (R&D Systems), and B-27supplement (Gibco). The number and size of spheres formed were evaluated under microscopy after 10 days. All experiments were performed in triplicate.

### *In vivo* proliferation and metastasis assay

Female BABL/c nude mice (4–5 weeks old) were purchased from the Experimental Animal Center of Guangdong Province (Guangzhou, China). All animal studies were conducted in accordance with the National Institutes of Health's animal use guidelines and current national regulations and standards regarding the use of laboratory animals. All animal procedures were approved by the Sun Yat-sen University Institutional Animal Care and Use Committee. For tumor growth assays, OVCAR-3 cells stably overexpressing miR-26b or scramble miRNA were resuspended in PBS, and 5 × 10^5^ cells (200 μl) were subcutaneously injected into the dorsal flank of nude mice. Tumor size was measured every 3 days and tumor volumes were calculated with following formula: volume = (L × S^2^)/2, in which L refers to the longest diameter and S shortest. Mice were sacrificed post three weeks, and tumors were dissected and weighted.

To investigate the effect of miR-26b on metastasis, OVCAR-3 cells stably overexpressing miR-26b or scramble miRNA (1× 10^6^ cells/mouse) were injected into the tail vein of nude mouse (six for each cell group). Six weeks after injection, the animals were sacrificed, and their lungs were dissected and paraffin-embedded. Consecutive sections (5 μm) were stained with hematoxylin and eosin. The micrometastases in the lungs were counted under a dissecting microscope. To detect the expression of KPNA2 and OCT4 *in vivo*, we carried out immunohistochemistry analysis according to standard procedures.

### Statistical analysis

Statistical analysis was performed using the SPSS software package (version 13.0, SPSS). Data is presented as the means ± SD and was assessed using a two-tailed Student's *t*-test. A threshold for statistically significant differences was set at *P* < 0.05. Regression analysis (Pearson's correlation) was used to assess the correlation of miR-26b and KPNA2 expression.
